# Sub-kb Hi-C in *D*. *melanogaster* reveals conserved characteristics of TADs between insect and mammalian cells

**DOI:** 10.1038/s41467-017-02526-9

**Published:** 2018-01-15

**Authors:** Qi Wang, Qiu Sun, Daniel M. Czajkowsky, Zhifeng Shao

**Affiliations:** 1 Shanghai Center for Systems Biomedicine, Shanghai Jiao Tong University, 200240 Shanghai, China; 2 State Key Laboratory for Oncogenes and Bio-ID Center, School of Biomedical Engineering, Shanghai Jiao Tong University, 200240 Shanghai, China

## Abstract

Topologically associating domains (TADs) are fundamental elements of the eukaryotic genomic structure. However, recent studies suggest that the insulating complexes, CTCF/cohesin, present at TAD borders in mammals are absent from those in *Drosophila melanogaster*, raising the possibility that border elements are not conserved among metazoans. Using in situ Hi-C with sub-kb resolution, here we show that the *D*. *melanogaster* genome is almost completely partitioned into >4000 TADs, nearly sevenfold more than previously identified. The overwhelming majority of these TADs are demarcated by the insulator complexes, BEAF-32/CP190, or BEAF-32/Chromator, indicating that these proteins may play an analogous role in flies as that of CTCF/cohesin in mammals. Moreover, extended regions previously thought to be unstructured are shown to consist of small contiguous TADs, a property also observed in mammals upon re-examination. Altogether, our work demonstrates that fundamental features associated with the higher-order folding of the genome are conserved from insects to mammals.

## Abstract (web summary)

Topologically associating domain (TAD) boundaries in flies seem to be different from those in mammals. Here, the authors use Hi-C with sub-kb resolution to identify about 4000 TADs in flies, most demarcated by the insulator complexes BEAF-32/CP190 or BEAF-32/Chromator like CTCF/cohesin in mammals.

## Introduction

It is now widely recognized that the three-dimensional (3D) structure of the genome plays a fundamental role in many nuclear processes, from cellular differentiation to transcriptional regulation to DNA replication and repair^1–6^. Methods derived from chromosome conformation capture (3C)^7^, such as 5C^8^ and Hi-C^9^, have proven particularly instrumental in this regard, revealing topologically associating domains (TADs) within which genomic loci are found to contact each other more frequently than those between adjacent TADs or in adjacent de-condensed, unstructured “inter-TAD” regions between TADs. Such TADs and inter-TAD regions have now been observed in most eukaryotic cells, suggesting that these are basic structural elements of the genomic architecture^10,11^.

In mammalian cells, there are now a number of studies demonstrating that the insulator protein CTCF and cohesin co-localize to the borders of many TADs^12–14^, with CTCF directly interacting with specific DNA sequences and cohesin mediating long-range chromosomal interactions^13^. Based on studies of targeted deletion of specific CTCF binding sites, the presence of CTCF and cohesin at TAD borders has been shown to be pivotal for the formation of TADs^14–17^. However, whether these proteins or their homologs play a similar function in other metazoan cells is not clear. In particular, for the model organism *D. melanogaster*, recent Hi-C studies failed to demonstrate a significant enrichment of either dCTCF (the CTCF homolog in *D*. *melanogaster*) or cohesin at TAD borders^18,19^. Such a discrepancy is perplexing as one might expect such functionally important complexes to be conserved, given the overall conservation of many basic biological and physiological processes between mammals and *D*. *melanogaster* ^20^.

Here we re-investigate the global structure of the *D*. *melanogaster* genome using in situ Hi-C at high depth to achieve a restriction-site limited “map resolution” of ~200 bp. At this higher resolution, we find that there are many more (nearly sevenfold) TADs resolvable in this genomic structure than previously identified. More importantly, there is a strikingly high enrichment of pairs of the insulator proteins, BEAF-32/CP190, or BEAF-32/Chromator, at the TAD borders, analogous to the enrichment of CTCF/cohesin pairs at TAD borders in mammalian cells. Further, while most of the previously identified TADs, primarily enriched for inactive chromatin, are now resolved as higher-order assemblages of smaller TADs, unexpectedly, the previously identified inter-TAD regions, thought to be unstructured, are actually composed of a string of well-defined small TADs with limited near-range inter-TAD contacts, a feature that can also be identified in mammalian cells. Taken together, these results strongly suggest that several of the most basic features of the higher-order genome architecture are conserved from insects to mammals.

## Results

### The fly genome is fully partitioned into contiguous TADs

Since the chromosome structure in eukaryotic cells is known to change significantly during the cell cycle^21^, we sought to minimize the variability in our examination of the genomic structure of the model eukaryote, *D*. *melanogaster*, by studying cells that were arrested at the G1/S boundary. To this end, we incubated S2R+ cells^22^, a well-studied cell line derived from the late embryo, with hydroxyurea, which is an effective inhibitor of eukaryotic DNA replication^23^ (Supplementary Fig. 1). We performed in situ Hi-C using the 4-cutter restriction enzyme, DpnII, following an established protocol^24^ with minor changes (Methods, Supplementary Methods). The median length of the DpnII restriction fragments in this genome is 194 bp. Sequencing the Hi-C library generated 695 million raw reads, which yielded 255 million high-quality read-pairs after all filtration steps (Supplementary Methods). To evaluate the reliability of this data, we also performed in situ Hi-C on a biological duplicate, sequencing to a lower depth of 253 million raw reads that yielded 98 million valid pairs. The two data sets were highly correlated (Pearson’s correlation, *r* = 0.98) (Supplementary Methods). Consequently, for all further analysis, we combined both data sets to finally obtain 353 million pair-end reads with a maximal estimated “map resolution” of ~200 bp, as calculated following Rao et al.^24^

To ensure the validity of our data, we generated a contact map at a lower resolution (20 kb) and compared it with that obtained previously from the highly related, S2 cells that was of this resolution^19^. Using the Armatus software to annotate TADs^19,25^, we identified 612 TADs that exhibited a median size of 140 kb, bordered by inter-TAD regions of a median size of 40 kb (Supplementary Data 1). These results are in excellent agreement with this earlier study^19^. In fact, the precise location of our TAD borders exhibited a high degree of overlap (81.3%) with those identified in the previous work (Supplementary Methods, Supplementary Fig. 2a and b). We also confirmed the lack of significant co-localization of dCTCF or cohesin at these TAD borders (Supplementary Methods, Supplementary Fig. 2c). Thus, at this lower resolution, our data and analysis agree substantially with this earlier study.

**Fig. 1:**
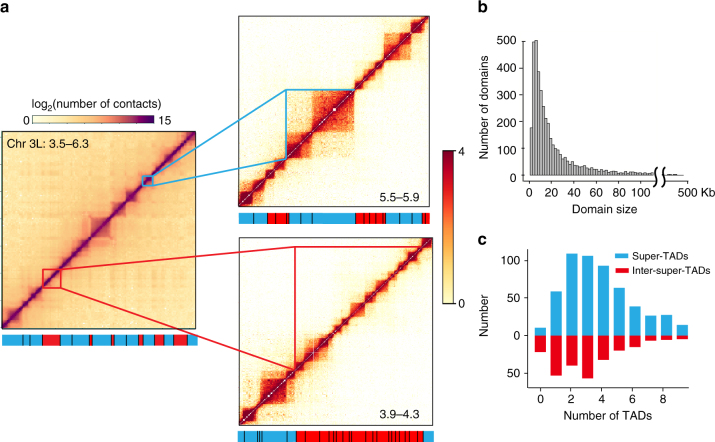
The *Drosophila* genome is fully partitioned into contiguous TADs including within previously annotated “inter-TADs” regions. **a** Heatmaps from the left arm of chromosome 3. The left panel shows a heatmap of a 2.8 Mb region of this arm at 20 kb resolution, revealing well-defined super-TADs (blue bars at the bottom) and inter-super-TADs (red bars at the bottom), consistent with previous findings^19^. At higher resolution (~200 bp), the heatmap shows that both the super-TADs (right upper panel) and the inter-super-TADs (right lower panel) are composed of small contiguous TADs. The blue (red) bars in these panels now refer to TADs within the super-TADs (inter-super-TADs). **b** The size distribution of the TADs annotated from the fragment-limited resolution map. The median size of the TADs is 13 kb. **c** The number of TADs within the super-TADs (blue bars) and inter-super-TADs (red bars)

However, when our data are examined at the higher, restriction fragment-limited resolution, it is immediately apparent that there are in fact many small TADs within both previously defined TADs and, notably, within the so-called “inter-TAD” regions (Fig. 1a). To avoid confusion, we will henceforth refer to those TADs identified at 20 kb resolution as “super-TADs” as they are in general much larger than those observed at the fragment-limited resolution, which we will refer to as “TADs.” Likewise, we will refer to the regions between the super-TADs as “inter-super-TADs.”

In total, we identified 4123 TADs that range in size from 3 to 460 kb, with a median size of 13 kb, that altogether cover almost the entire (>92%) 130 Mb non-repetitive *D*. *melanogaster* genome (Fig. 1a, b, Supplementary Fig. 3). As shown in Fig. 1, the super-TADs are now found to be subdivided into most frequently 2-4 small TADs (median size, 16 kb, Fig. 1c). By contrast, the inter-super-TADs that were previously considered largely devoid of identifiable organization are shown to completely consist of generally 1–4, slightly smaller TADs (median size, 9 kb, Fig. 1c).

A striking feature of the distribution of these TADs, whether associated with a super-TAD or inter-super-TAD, is that most (75.4%) of the borders between adjacent TADs localize to the same restriction fragment (Supplementary Fig. 3, Supplementary Data 2). That is, at the resolution limited by the size of the restriction fragments, the TADs are essentially contiguous, without an unstructured region in between, illustrating that, unlike what was concluded from lower resolution maps, there are essentially no extended stretches of “inter-TADs” across the genome.

### Demarcation of TADs by specific pairs of insulator proteins

**Fig. 2:**
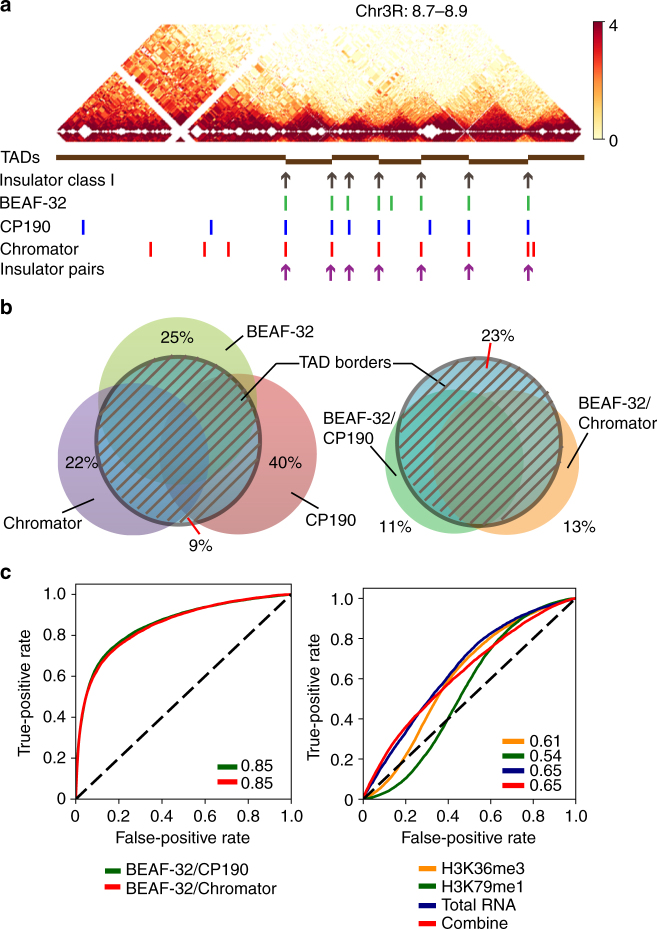
The TADs are demarcated by pairs of insulator proteins. **a** The locations of known *Drosophila* insulator proteins, together with the TADs identified in this work, are shown for a 200 kb segment of chr3R. Shown are the positions of class I insulator proteins (that includes BEAF-32 and CP190), as obtained from Flybase, as well as the peak locations of the individual insulator proteins (BEAF-32, CP190, and Chromator) characterized in the modENCODE project. Also shown in the bottom row are the positions of the insulator protein pairs, BEAF-32/CP190, or BEAF-32/Chromator. **b** Venn diagram showing the genome-wide co-localization of these insulator proteins and insulator protein pairs at the TAD borders. **c** Logistic regression models to examine the predictive power of the insulator protein pairs (left panel) or transcriptionally active epigenetic modifications or transcriptional levels (right panel) of TAD borders

Yet, even with these more precisely defined borders, a comparison with the known locations of dCTCF or cohesin subunits showed an absence of significant enrichment at TAD borders (Supplementary Fig. 4a), consistent with previous work showing that neither protein defines border elements in *D*. *melanogaster* ^18,19^. However, since this organism contains many other insulator proteins^26,27^, we reasoned that other insulator proteins might function as analogs of CTCF/cohesin in this organism instead. An early study identified two classes of insulator proteins in *Drosophila* embryos^28^: Class I (that includes BEAF-32 and CP190) and class II (that includes only Su(Hw)). Surprisingly, using the binding site locations defined in this earlier work, we found an exceptionally high co-localization of class I insulator proteins at these narrowly defined TAD borders (Fig. 2a, Supplementary Fig. 4b). By contrast, the class II insulator protein was not significantly associated with TAD borders (Supplementary Fig. 4b and c).

To determine the enrichment of individual insulator proteins, we analyzed the location of all insulator proteins profiled in the modENCODE project in S2 cells (namely, BEAF-32, Chromator, CP190, dCTCF, GAF, mod(mdg4), Su(Hw), and ZW5). We found that BEAF-32, Chromator, and CP190 are each significantly enriched at the boundaries of the TADs (Supplementary Fig. 4a *)* while no other insulator protein exhibits such significant enrichment at these TAD borders (Supplementary Fig. 4a). Overall, >91% of all TAD borders contain at least one of these three proteins (Fig. 2b), an enrichment that far exceeds what would be expected from a random distribution (Fisher’s exact test, *p* value < 2.2e−16).

However, we also found that, as with CTCF and cohesin in mammalian cells^13,14,29^, each of these insulator proteins is found at many other locations in addition to TAD borders (Fig. 2b). Since previous work has shown that BEAF-32 binds to specific DNA sequences^26,30^ and CP190 and Chromator both bind to BEAF-32 and mediate long-range chromosomal contacts^30^, we examined if there is a greater degree of exclusivity at the TAD borders of pairs of insulator proteins (BEAF-32/CP190 or BEAF-32/Chromator) than what is observed with the individual proteins. Indeed, we found that 74% of pairs BEAF-32/CP190 or BEAF-32/Chromator localize to the TAD borders, and conversely, 77% of the borders localize to the binding sites of these pairs (Fig. 2b). This striking correlation holds true over a wide range of the Armatus TAD annotation parameters (Supplementary Fig. 4d).

We further validated this enrichment by examining the extent to which the positions of these protein pairs alone could predict the location of TAD borders using logistic regression, as described in previous work^19^. We found that regression based on the locations of the pairs of BEAF-32/CP190 or BEAF-32/Chromator is highly predictive of a TAD border (Fig. 2c). By contrast, similar analysis with active transcription markers (H3k26me3 and H3k79me1) or total RNA or their combination, which have been previously suggested to be generally associated with TAD boundaries in *Drosophila* ^19^, are substantially less predictive of TAD borders (Fig. 2c). Thus, BEAF-32/CP190 and BEAF-32/Chromator may be defined as bona fide TAD border elements in *D*. *melanogaster*.

### Chromatin state and inter-TAD interactions

**Fig. 3:**
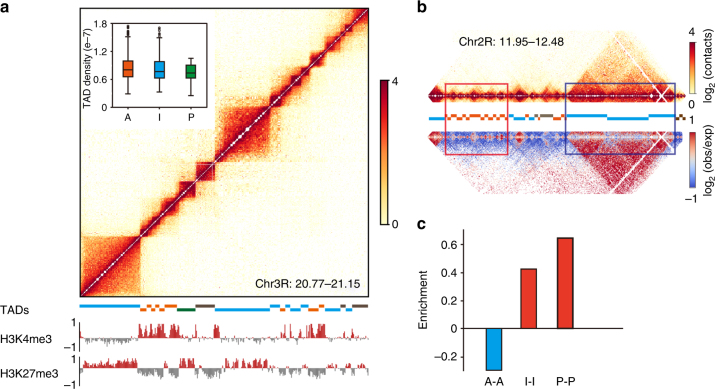
Epigenetic modifications only correlate with higher-order folding of the TADs but not the folding of individual TADs. **a** The TADs could be classified into four major types according to the enrichment of 15 histone modifications and non-histone chromosomal proteins within each TAD (Supplementary Methods). Shown is an example of the distribution of these types with active (orange bar below the heatmap), inactive (blue bar), polycomb (green bar), and undetermined (gray bar) chromatin within the TADs in a 380 kb region of chr3R. Inset: the extent of DNA condensation within the TADs, as determined from the average of contact frequencies between loci within the TAD. **b** Comparison of the frequency with which active or inactive TADs tend to interact with their immediately neighboring TADs. The upper heatmap shows the positions of the TADs, while the lower heatmap shows the significance of the observed contacts, with those colored red (blue) exhibiting much greater (lower) interaction strength than expected by chance (Methods) in a 530 kb region of chr2R. **c** The relative interaction strength (as shown in **b**) between pairs of TADs. A active, I inactive, P polycomb

Previous work suggested that histone modifications are a major driving factor for TAD formation in *Drosophila* and other eukaryotes^19,31^. To examine the relationship between chromatin state and the TADs identified here, we first classified the TADs according to the enrichment of 15 histone modifications and non-histone chromosomal proteins within each TAD using *k*-means clustering^32^, identifying eight different types that could be broadly grouped into four major types of TADs: those enriched with active, inactive, or polycomb-associated chromatin marks/proteins, and those without any of these features (“undetermined”) (Fig. 3a, Supplementary Fig. 5a and b). Consistent with previous work, we found that 83% of TADs enriched for inactive chromatin localize within super-TADs, while 81% of TADs enriched for active chromatin localize within inter-super-TADs, a highly non-random distribution (Fisher’s exact test with *p* value < 2.2e−16). We note, though, that such a correlation is not present at the TAD level.

However, we found that, at least over the distances over which a comparison can be made (Methods, Supplementary Fig. 5c), the TADs enriched for inactive chromatin exhibit the same level of DNA condensation as those enriched for active chromatin, as determined from the average contact frequencies within TADs of comparable size, as previously^19^ (Fig. 3a, inset). By contrast, there is a significant enrichment of inactive–inactive or polycomb–polycomb inter-TAD contacts between neighboring TADs and a strong depletion of active-active TAD contacts over what would be observed by chance (Fig. 3b, c; see Supplementary Fig. 6 for the length distribution). Further, overall, the TADs within the super-TADs make far more frequent contacts with other TADs within the super-TAD, while those within inter-super-TADs contact each other and make significantly fewer contacts with neighboring TADs (Supplementary Fig. 7). Thus, overall, this analysis indicates that the chromatin state may be a contributing factor not for condensation within TADs, but rather for interactions between immediate neighbors of TADs responsible for the folding into the higher-order super-TAD structures.

### Conserved features between *D*. *melanogaster* and mammals

**Fig. 4:**
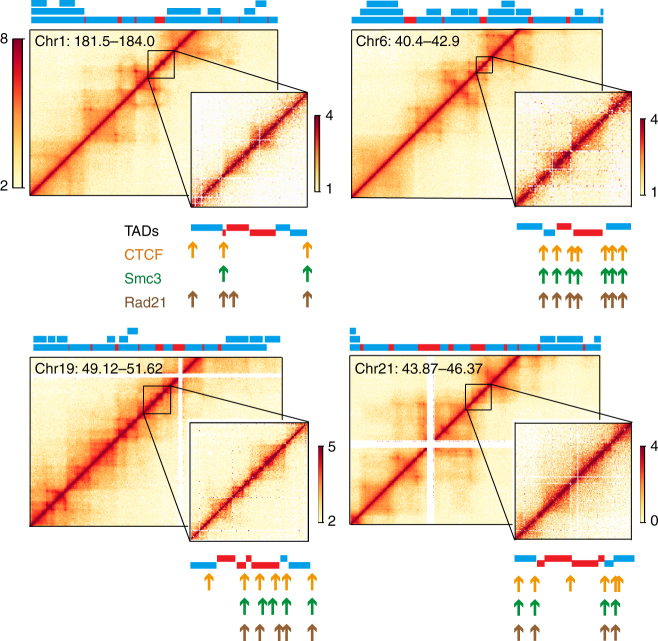
The human genome is also partitioned into contiguous small TADs within previously described “inter-TAD” regions at least in part. Shown are four examples of Hi-C data of GM12878 lymphoblastoid cells determined by Rao et al.^24^ In each panel, the main figure is the heatmap of the indicated chromosomal position at 5 kb resolution, with the domains annotated by these authors indicated by the color bars above each figure. Note that there were smaller TADs within larger TADs identified in this previous work, reflected in the three different levels in the annotated TADs. The bars colored red reflect the inter-TAD regions, while those colored blue are the TADs. The inset of each panel is an expanded region of an inter-TAD region. The TADs annotated using the Armatus software are shown below the heatmap. Also shown are the locations of CTCF (orange arrows) and cohesin components (*Rad*21 and Smc3, green and brown arrows. respectively), as determined previously^24^

An unmistakable feature of the genomic structure revealed by this high-resolution map is that essentially all of the genome is folded into TADs, with more highly ordered super-TADs separated by open regions of smaller TADs. To determine whether these structural details are only characteristics of the fly genome, we sought for evidence of these features in previously studied mammalian cells. While, to our knowledge, there is no published Hi-C study of synchronized mammalian cells to the resolution in our work, we re-examined the Hi-C data from asynchronous human lymphoblastoid (GM12878) cells with 1 kb resolution^24^. As shown in Fig. 4, there were indeed many small (median size, 34 kb) contiguous TADs readily identifiable within previously defined inter-TADs. A reanalysis of chromosome 1 from this mammalian data set identifies roughly one half of all previously defined inter-TADs to contain one or more small TADs (Supplementary Fig. 8). Some of the borders of these smaller TADs are also bound by CTCF/cohesin (Fig. 4). This earlier work noted several occurrences of larger TADs that, like the super-TADs found in *Drosophila*, are composed of smaller TADs^24^. Thus, the general TAD-level organization observed in *D*. *melanogaster* may also be a conserved feature of the genomic structure of mammals as well.

## Discussion

Using in situ Hi-C with ~200 bp resolution, we have examined the 3D organization of the *D*. *melanogaster* genome. We have found that this genome contains many more TADs than previously thought, most of which are smaller than what could be resolved in previous Hi-C studies. What emerges from an analysis of this high-resolution map is that the genome structure generally consists of alternating stretches of two different types of TADs that differ slightly in size (9 vs 16 kb), but differ more significantly in the degree to which the TADs engage in inter-TAD interactions, with those within the super-TADs making more extensive inter-TAD contacts. These self-associating TADs within the super-TAD are highly enriched for inactive chromatin, while the weakly interacting TADs within the inter-super-TADs are predominantly enriched for active chromatin. We also performed Hi-C with asynchronous cells, finding similar results (though with less well-defined TAD borders, Supplementary Methods, Supplementary Fig. 9a and b). Further, we re-analyzed data from a recently published Hi-C study of asynchronous Kc167 cells^18^, and found generally similar results (Supplementary Methods, Supplementary Fig. 10a and b). Thus, these are basic structural properties of the genome of *Drosophila* cells, regardless of cell cycle stage.

The finding that the inter-super-TAD region is completely partitioned into contiguous small TADs is highly unexpected based on previous Hi-C studies in both *Drosophila* and mammalian cells^10,33,34^. In these, this unstructured open chromatin organization was suggested to be a consequence of active chromatin and active transcription, which was suggested to be generally inhibitive of well-organized genomic structures^10,19^. However, our findings strongly suggest that chromatin state does not play a primary role in the formation of TADs: (i) the inter-super-TAD region in fact contains many distinct TADs (Fig. 1a); (ii) active chromatin and active transcription are not highly predictive of TAD borders (Fig. 2c); (iii) these TADs are not solely enriched for a single epigenetic type (Supplementary Fig. 5a); and (iv) the TADs enriched for a specific chromatin state are found to exhibit a significant degree of heterogeneity of the modification contained within (Supplementary Methods, Supplementary Fig. 11). While the underlying mechanisms responsible for TAD formation remains to be determined and may differ between active and inactive chromatin, our results are consistent with chromatin state influencing higher-order folding of the TADs into super-TADs. Further, our findings instead suggest that components at the TAD border may play a more determinative role in TAD formation, since regardless of the chromatin type contained therein, the chromatin is similarly compacted (Fig. 3a, inset).

Our work also provides convincing evidence that the defining feature of the overwhelming majority of the TAD borders is the co-localization of pairs of specific insulator proteins, BEAF-32/CP190, or BEAF-32/Chromator. These pairs are enriched at all TAD borders, regardless of the type of chromatin contained within the adjacent TADs (Supplementary Methods, Supplementary Fig. 12). There are many other insulator proteins in this organism^26,27^, but none show significant enrichment at these borders (Supplementary Fig. 4a). Thus, the enrichment of insulator proteins at TAD borders is not a general property of all insulator proteins in this organism, and thus, probably, of insulation per se. Conversely, we speculate that it is the presence of these pairs of insulator proteins that determines the presence of a TAD border.

The presence of a pair of proteins at the TAD border, one of which specifically binds DNA and the other that mediates long-range chromosomal interactions, which we observe here in *D*. *melanogaster*, is strikingly similar to what is observed at many TAD borders in mammalian cells with CTCF/cohesin, suggesting that BEAF-32/CP190 and BEAF-32/Chromator are functional analogs of CTCF/cohesin as TAD border elements. Since knockdown of BEAF-32 leads to severe cell cycle defects^35^, further delineation of its function will require a more detailed determination of its DNA binding site, which remains ill-defined^26,30,36–38^.

We also note though that, consistent with previous Hi-C studies in *Drosophila* ^18,19,33,34,39–41^, most TAD borders in *Drosophila* are not associated with enriched contact frequency (“corner peaks”) common at many CTCF/cohesin TAD borders in mammalian cells^24^. Whether their absence in the *Drosophila* data is a technical issue related to the significantly smaller TAD size in the fly^42^ or to unknown mechanisms remains to be further delineated. In addition, since CTCF and cohesin are not found at all TAD borders in the mammalian genome, there may be other similar protein pairs^43^ that are also functionally analogs of CTCF/cohesin at these other TAD borders in mammalian cells. Recent work has indeed identified many other “architectural” proteins in mammals, some of which appear to be enriched in some TAD borders^44,45^.

We have also noted other structural details of TADs that may be conserved between *D*. *melanogaster* and mammals, most notably that the genome structure may generally consist of alternating stretches of self-associating small TADs (see also ref. ^42^) and weakly associating small TADs. The conservation of this particular pattern likely reflects necessary functional utility, perhaps providing the combination of order yet flexibility needed for a range of genomic functions^4,46^.

In conclusion, *Drosophila* is considered as a model eukaryote whose study provides direct information of basic biological processes in higher-level organisms^20,47^. Our work here extends these similarities to that of genome structure, which further underscores its important role in many fundamental genomic processes. While, as suggested here, the precise molecular components generating this structure may be different, the underlying basic structural features appear to be well conserved. Future work designed to characterize the physical and dynamic details of these basic features may eventually lead to an understanding of the underlying mechanisms in the various genomic processes that are conserved from insects to mammals.

## Methods

### Cell culture and synchronization

*Drosophila* late embryonic S2R+ cells (DGRC, stock number: 150) were grown in Schneider’s medium (Invitrogen) with 10% heat-inactivated fetal bovine serum (BI) at 25 °C. Cells were synchronized at G1/S by incubating with 1 µM hydroxyurea for 18 h^23^.

### Hi-C library preparation and data processing

Hi-C libraries of two biological replicates for both asynchronous and G1/S arrested cells were generated utilizing the in situ Hi-C method^24^ with minor modifications. Briefly, nuclei released from 10 million crosslinked cells were digested with DpnII (NEB). After end repair and ligation, the biotin-labeled chimeric molecules were fragmented with Cavrios M220, and the fragments between 300 and 500 bp were selected for the generation of library. The libraries were prepared using NEBNext Ultra DNA library prep kit (#E7370, NEB) according to the manufacturer’s instructions with minor modifications (Supplementary Methods). The libraries were then sequenced using the Illumina ×10 platform.

The Hi-C reads were iteratively mapped to the dm3 *D. melanogaster* reference genome using bowtie2 (v2.2.9) (Supplementary Methods). After filtering, the valid contact matrix was normalized using ICE as described^48^. After normalization, domains were annotated using the software Armatus^25^ with the scaling parameter, gamma, set to 0.9. The other parameters in Armatus for *Drosophila* data were: -R –N –g 2.0 –m –r 1 –s 0.1 (where here –r 1 refers to single fragment resolution). For the human data, they were: -R –N –g 2.0 –m –r 1000 –s 0.1. However, visual inspection of the analyzed data revealed that some domain borders were immediately adjacent to regions with no reads, suggesting that these locations may have been defined as borders owing to absence of reads in the adjacent region. Thus, we scanned through the analyzed data and, where there was a domain boundary adjacent to a read with no reads, we re-analyzed the data using a gamma value of 0.6.

For the calculation of the number of TADs within the super-TADs and inter-super-TADs, we established a threshold value of 75% of the domain length for inclusion in either a super-TAD or inter-super-TAD.

### Extent of enrichment of insulators at TAD borders

All peaks and normalized signal tracks for each insulator protein were obtained from the modENCODE database. For each domain boundary, we first identified the boundary center as the midpoint between the end position of the upstream domain and the start position of the downstream domain. For each insulator protein, we calculated the average occupancy value within 4 kb of each boundary center using an 80 bp window. We used the same method to calculate the background values by randomly changing the positons of the border centers over the entire genome. The ratios of values obtained from actual boundary centers to that from randomly shuffled centers were used to evaluate the enrichment of insulator proteins at the boundaries.

For the evaluation of the co-localization of insulator proteins with TAD borders, we considered any insulator protein peak localized within 2 kb of the domain boundary as co-localized with that boundary.

### Prediction of domain boundaries

We used the function linear_model.LogisticRegression from the Python package scikit-learn (v0.18) to implement a logistic regression model similar to that described^19^ to predict the domain boundaries using different combinations of epigenetic and insulator markers. The input variables were *Z*-transformed signals of different markers for each fragment from the modENCODE database, with an output value of 0 indicating an intra-domain fragment and a value of 1 indicating a border-related fragment. Training sets and test sets were separated randomly with equal sizes using the cross_validation.train_test_split function. The receiver-operating characteristic curves and area under curve values were calculated using the functions metrics.roc_curve and metrics.auc from scikit-learn.

### Determination of the DNA condensation within TADs

We calculated the average contact frequency of all pairs of fragments located inside each TAD as a measure of DNA condensation. The restriction fragments with no ligation products were removed from this calculation. To avoid complications arising from comparing domains of significantly different sizes, we compared only those domains of roughly the same size (~15 kb; range 5–20 kb), since for this range, there are a sufficient number of domains of each type.

### Calculation of the enrichment of TAD–TAD interactions

For the evaluation of TAD–TAD interactions, an enrichment ratio matrix was first calculated by dividing the contact number of each pair of fragments by the average contact number of all pairs of fragments that have the same interaction distance, binning the distances using a 200 bp window. An average enrichment ratio was then calculated for each pair of TADs by averaging all the enrichment ratios of all pairs of fragments localized in this pair of TADs.

### Data analysis of Hi-C data of human GM12878 cells

We downloaded the GM12878 Hi-C data from the GEO database with accession number GSE63525. We determined the normalized Hi-C heatmap using the KR normalization factors^24^. We used the Armatus software to annotate TADs in the 1 kb resolution data using a gamma value of 0.7. We also annotated large TADs at 5 kb resolution using different gammas (0.6–1.0) and the majority (70.9%) of boundaries of the TADs identified by Rao et al.^24^ were located within two bins of the Armatus domain boundaries.

### Data availability

All sequencing data that support the findings of this study have been deposited in the National Center for Biotechnology Information Gene Expression Omnibus (GEO) and are accessible through the GEO Series accession number GSE101317.

## Electronic supplementary material

Supplementary Information

Description of Additional Supplementary Files

Supplementary Data 1

Supplementary Data 2
