# Supplementary material for: Sub-kb Hi-C in D. melanogaster reveals conserved characteristics of TADs between insect and mammalian cells
Source: Nat Commun. 2018 Jan 15;9:188. doi: 10.1038/s41467-017-02526-9 (PMC5768742; doi:10.1038/s41467-017-02526-9)
Supplement: Supplementary file 1 — Supplementary Information [file 41467_2017_2526_MOESM1_ESM.pdf]

Supplementary Methods

In situ Hi-C Library Preparation

Hi-C libraries were generated using a standard *in situ* Hi-C protocol [1], with minor modifications described below. Ten million S2R+ cells were harvested, washed twice, re-suspended with 18 ml of Schneider's medium without FBS, and then crosslinked with 950 µl of 20% freshly prepared formaldehyde (final concentration 1%) for 10 minutes at room temperature while mixing. Formaldehyde was quenched by adding 1 ml of 2.5 M glycine (0.125 M final concentration) and incubating for 5 minutes at room temperature and then on ice for 20 minutes. The fixed cells were washed with ice-cold PBS, pelleted by centrifugation, flash frozen in liquid nitrogen and stored at -80 °C.

After thawing, the fixed cells were resuspended in 1 ml of lysis buffer (10 mM Tris-HCl pH 8.0, 10 mM NaCl, 0.2% Igepal CA-630, 1/10 vol. of proteinase inhibitor cocktail (Sigma)), and then incubated on ice for 20 minutes. Nuclei were pelleted by centrifugation at 4 °C, 600x g for 5 minutes, and then washed with 1 ml of the lysis buffer, followed by another centrifugation under similar conditions. After washing twice with 1.2X DpnII NEBuffer (New England Biolabs, NEB), the nuclei were resuspended in 400 µl of 1.2× DpnII NEBuffer. 5 µl of 10% SDS was then added to a final concentration of 0.1%, and the nuclei were incubated at 62 °C for 10 minutes. After, 41 µl of 10% Triton X-100 was added to a final concentration of 1%, and the nuclei were incubated for 20 minutes at 37 °C. The nuclei were digested overnight at 37 °C with 300 U of the restriction endonuclease, DpnII (10 U/µl, NEB) with slow rotation. The sample was then incubated at 62 °C for 20 minutes to inactive DpnII.

The nuclei were pelleted by centrifuge at 4 °C, 600x g for 5 minutes and washed twice with 1X NEBuffer II (NEB). The pellet were re-suspended with 475 µl of 1X

NEBuffer II, and then 52 µl of biotin fill-in master mix was added and mixed well (fill-in master mix: 1.5 µl of 10 mM dATP (Thermo Scientific), 1.5 µl of 10 mM dGTP (Thermo Scientific), 1.5 µl of 10 mM dTTP (Thermo Scientific), 37.5 µl of 10 mM biotin-14-dCTP (Invitrogen), 10 µl of Klenow enzyme (NEB)). The mixture was incubated at 37 °C for 60 minutes with slow rotation. After the biotin fill-in reaction, the nuclei were harvested by centrifugation at 4 °C, 600x g for 5 minutes, and re-suspended by adding 1 ml of the ligation master mix, pipetting gently to mix well, followed by incubation at 25 °C for 4 h (ligation master mix: 100 µl of 10X T4 ligase buffer (NEB), 100 µl of 10% Triton, 10 µl of 100X BSA, 750 µl of autoclaved deionized water, 10 µl of T4 ligase (NEB)).

The crosslinking was reversed by overnight incubation at 65 °C in the presence of proteinase K (100 µg/ml) and NaCl (0.5M). After reversal, the DNA was purified by extraction with 2 vol. of phenol-chloroform followed by ethanol precipitation. After precipitation, the pellets were dissolved in 200 µl of 10 mM Tris-HCl pH 8.0, followed by the addition of 2 µL of 20 mg/mL RNase A (Invitrogen) and incubation at 37 °C for 30 minutes. The DNA concentration was measured using the Qubit dsDNA HS Assay.

Two µg of DNA was diluted into 130 µl, followed by shearing using Covaris M220 Focused-ultrasonicator (Target length 400 bp, Peak Incident Power 50W, Duty Factor 10%, Cycles per Burst 200). The DNA fraction in the size range of 300-500 bp was selected using Agencourt AMPure XP beads (Beckman Coulter). Briefly, 130 µl of the DNA solution was transferred into a new 1.5 ml low-binding tube, and the Covaris tube was washed with 70 µl of 10 mM Tris-HCl, which was transferred into the same low-binding tube. Then, 110 µl of completely re-suspended AMPure XP beads (maintained at room temperature for at least 30 minutes prior to use) were added and mixed by vortexing. After incubation at room temperature for 5 minutes, the beads were collected with a magnet, and the supernatant was transferred to a fresh tube. Thirty µl of XP beads were added to the supernatant, vortexed, and then incubated at room temperature for 5 minutes. The beads were collected with a

magnet and the supernatant was discarded. The beads were then washed three times while still associated with the magnet with 300 µl of freshly prepared 80% ethanol and then air dried. The beads were then re-suspended with 60 µl of 10 mM Tris-HCl pH 8.0 and incubated at room temperature for 5 minutes. The beads were then collected with the magnet, and 57 µl of the DNA eluate was transferred into a fresh tube. The DNA concentration was determined with a Qubit dsDNA HS Assay.

DNA was end-repaired, 'A'-tailed and ligated with Illumina compatible adapters using the "NEBNext End Prep" and "Adaptor ligation" module in the NEBNext Ultra DNA Library Prep Kit for Illumina (NEB) according to the manufacturer's instructions. If more than 1 µg of DNA was obtained, multiple reactions were performed in parallel. After the ligation reaction was completed, the mixture was transferred into a new 1.5 ml low-binding tube, and autoclaved water was added for a total volume of 300 µl.

Biotin-mediated pull-down of the ligation junctions was performed as described previously (Rao et al, 2014) with minor modifications. Briefly, 20 µl of MyOne Dynabeads Streptavidin C1 (Invitrogen) beads were used to capture 1 µg of the biotinylated DNA. The C1 beads were washed twice with 400 µl of Tween Washing Buffer (TWB, 5 mM Tris-HCl pH 7.5, 0.5 mM EDTA, 1 M NaCl, 0.05% (v/v) Tween) and then re-suspended in 300 µl of 2x Binding Buffer (BB, 10 mM Tris-HCl pH 7.5, 1 mM EDTA, 2 M NaCl). Adaptor-ligated DNA was added to the beads and incubated at room temperature for 20 minutes with slow rotation. Beads were collected with a magnet, the supernatant discarded, the beads washed twice with 400 µl of TWB at 50 °C for 2 minutes, and then washed twice with 100 µl of 10 mM Tris-HCl pH 8.0.

The DNA was eluted by re-suspending the beads with 24 µl 10 mM Tris-HCl pH 8.0, and incubating at 98 °C for 10 minutes. The beads were then collected with a magnet and 23 µl of the DNA suspension was transferred into a fresh PCR tube. One µl of index primer, 1 µl of universal PCR primer and 25 µl of NEBNext High Fidelity 2X

PCR Master Mix were then added into the PCR tube. After mixing by pipetting, the PCR reactions were performed following the manufacturer's instructions (NEBNext Ultra DNA Library Prep Kit for Illumina (NEB)). The PCR cycling conditions were: step 1, initial denaturation 98 °C for 30 seconds; step 2, denaturation 98 °C for 10 seconds; step 3, annealing 65 °C for 30 seconds; step 4, extension 72 °C for 30 seconds; go to step 2 for another 7 cycles; final extension 72 °C for 5 minutes, hold at 4 °C.

The quality of the Hi-C libraries was determined using the Qubit dsDNA HS Assay and Agilent 2100 DNA 1000 HS kit. The high-quality libraries were sequenced with an Illumina X-ten platform with paired-end 150 bp reads.

Hi-C data processing and TAD annotation

Owing to the long length of our reads (150 bp), it is possible that there are many chimeric reads in which the ligation junction is located within one end. To solve this problem, we used an iterative method for Hi-C mapping: For each end of the Hi-C reads, we first used a length of 25 bp to map; if this region failed to map uniquely, we included an additional 25 bp in the next round of mapping. We continued this procedure until the full read length reached 150 bp. The Hi-C reads were mapped to the dm3 *Drosophila melanogaster* reference genome using bowtie2 (v2.2.9). We only kept read pairs for which the mapping quality (MAPQ) for each end is larger than 30. We further removed reads mapped to the same restriction fragment and dangling reads that were separated by less than 500 bp, which is the library selection size and PCR duplicates. The two biological replicates, one from each of the G1/S synchronized and asynchronous cells, were processed using the same procedure. To test the validity of our data, we calculated the Pearson correlation coefficient between each pair of replicates, between the merged G1/S and asynchronous datasets and between the merged asynchronous dataset and the previous published S2 dataset [2] after scaling to the same read depth (100 million).

We normalized our Hi-C contact maps using ICE as described [3]. Instead of using bins with a fixed size, we treated each restriction fragment as one unit and counted the total number of contacts occurring between each pair of restriction fragments. We assumed that the total contacts for each fragment are equal. Analyzing the data in this way provides the highest possible resolution and, further, using fragments instead of equally spaced bins also avoids problems resulting from the lack of restriction sites inside fixed bins of a small size. The heatmap was generated using matplotlib in python.

After normalization, domains were annotated using the software Armatus [4] with scaling parameter, gamma, set to 0.9. However, visual inspection of the analyzed data revealed some domains whose boundaries appeared to result from the presence of regions with no reads. These domains were thus modified locally using gamma value of 0.6.

For the calculation of the number of TADs within the super-TADs and inter-super-TADs, we established a threshold value of 75% of the domain length for inclusion in either a super-TAD or inter-super-TAD.

TAD clustering using k-means

We used a representative set of 15 histone modification and protein markers to classify our TAD domains. The localization data for these modifications/proteins were obtained from the modENCODE database. Of these markers, H3K4me3, H3K4me2, H2B(ubiq), H3K79me2 and H3K36me3 are associated with active promoters and transcription elongation regions; H4K16ac and MSL-1 are primarily associated with X chromosome-related active regions although they also localize to active regions on other chromosomes; H3K4me1, H3K27ac and LSD1 are associated with active enhancer regions; H3K9me3, H3K9me2 and Su(var)3-9 are heterochromatin markers;

and H3K27me3 and Pc are associated with polycomb regions. We calculated the average ChIP signal strength for each of these modifications/proteins within the innermost region between 10% to 90% of the TAD domain length. We then used k-means clustering (using the KMeans function in scikit-learn) to classify our domains. The number of clusters, k, was determined by the silhouette score, yielding a value of 8. We performed this clustering procedure 500 times and chose the best result according to the clustering score. To test our k-means outcome, we also examined the data using hierarchical clustering and we obtained similar results. Based on the modification/protein characteristics described above, we characterized the first 4 clusters as active domains, cluster 5 and 6 as inactive domains, cluster 7 as polycomb domains and the last one as undetermined.

Insulation score calculation

In order to quantify the insulation strength for each domain border, we calculated the insulation score (see Fig S1B) at each border similar to the Directionality Index [5] using:

$$S_{i,i+1} = \frac{\left(D_i - E_{i,i+1}\right)^2}{E_{i,i+1}} + \frac{\left(D_{i+1} - E_{i,i+1}\right)^2}{E_{i,i+1}} \qquad (1)$$

where D_i is the averaged contacts number of all pairs of fragments located within the upstream domain of each domain border, D_{i+1} is the averaged contacts number of all pairs of fragments located within the downstream domain, and $E_{i,i+1}$ is the averaged contacts number of all pairs of fragments located within the larger domain merged by the upstream and downstream domains (see Supplementary Fig. 1b for the definitions). N is the total TAD number. A larger insulation score reflects a greater insulation strength of the domain border.

Epigenetic entropy

We used entropy to evaluate the heterogeneity of the distribution of epigenetic markers within each TAD, choosing 5 common active and inactive (H3K4me3, H3K27ac, H3K9me3, H3K9me2, H3K27me3) histone modification markers. First we transformed the Z-normalized signals for each epigenetic marker into positive values using

$$\mathrm{N} = e^{z} \qquad (2)$$

where Z is the Z-normalized signals. Then, we partitioned each TAD into 10 equally spaced intervals and calculated the fraction of the signals within each interval. We then calculated the entropy as:

$$\mathrm{E} = -\sum_{i=1}^{10}(p_i \times log_2 p_i) \qquad (3)$$

where p_i is the fraction of the transformed signals for each epigenetic marker localized into the ith interval.

PolyA+ RNA-seq library generation and data analysis

Total RNA were extracted from 2-5 million cells using Trizol Reagent (Invitrogen). The RNA quality was assessed using Agilent Bioanalyzer 2100. PolyA+ RNA-seq libraries were prepared using the KAPA Stranded mRNA-seq kit following the manufacturer’s instructions. After preparation, libraries were quantified using a

Qubit fluorometer and sequenced with Nextseq 500 (1 X 75bp).

Adapters and low quality reads were removed by cutadapt (v1.8.3) and Trimmomatic (v0.36) using default parameters. Valid paired reads were mapped to the *Drosophila melanogaster* reference genome dm3 and Flybase gene annotation v5.57 using Tophat2 (v2.0.9) with default options and transcripts were assembled using Cufflinks (v2.2.1). Two replicates (Pearson's correlation, r = 0.91) were merged for further analysis. The coverage of RNAseq reads over the whole genome obtained using genomecov in bedtools (v2.22.1) was used to evaluate the predictive power by logistic regression.

Kc167 data re-analysis

The Hi-C data of Kc167 cells [6] were obtained from the GEO database with accession number GSE80702. To obtain the fragment resolution map, we only used Hi-C libraries built using the restriction enzyme, HinfI, instead of combining all the libraries built with different restriction enzymes, since we found that the HinfI data has more clear domain structures at fragment resolution than the combined data at 1kb resolution. In addition, using fragment-resolution limited bins instead of 1kb bins achieved similar resolution (220 bp) as our DpnII data. We performed the same Hi-C analysis procedure as described above for the S2R+ data and used Armatus to call domains at fragment resolution. We first used a gamma value of 1.0 and corrected the domains using a gamma value of 0.7, as described above.

The ChIP-seq data of the insulator proteins for Kc167 were obtained from the GEO database with accession number GSM762845 (BEAF-32), GSM762836 (CP190) and GSM1535975 (Chromator). We mapped all the reads to the *Drosophila melanogaster* dm3 reference genome using Bowtie2 (v2.2.9) and we used MACS (v1.4.2) to call peaks with default parameters using a p value cutoff 1e-5.

When calculating the overlap of borders between Kc167 and S2R+ cells, we defined the border region as the region from 1 kb upstream to 1 kb downstream of the restriction sites.

Long-range interactions

Significant interactions between loci were identified using Fit-Hi-C (v1.0.1) using default parameters at 1 kb resolution with q-values ≤ 0.01. Intra-domain interactions were not included in these calculations. Interacting loci were examined according to the chromatin state with which their TAD is associated. The length distribution of the significant frequencies was then smoothed using Gaussian Kernel smoothing using the kdeplot function (with parameters bw=100) in the python package Seaborn (v0.7.1)

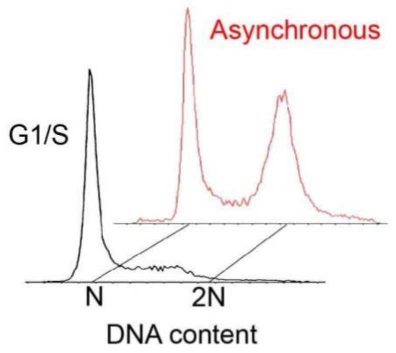

Supplementary Figure 1. Cell culture synchronization. FACS analysis showing the DNA content of asynchronous and G1/S arrested S2+ cells.

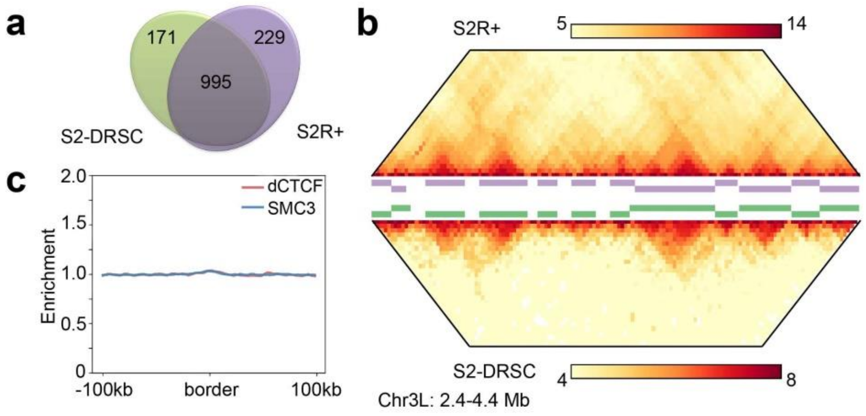

Supplementary Figure 2. Validation of the data by examination of the heatmap at 20 kb resolution. (a) The TADs annotated in our 20 kb resolution map from S2R+ cells are highly similar to those identified previously from S2-DRSC cells[2] (b) A comparison of a 2Mb region of chr3L from the S2R+ and S2-DRSC cells. (c) There is no significant enrichment of either dCTCF or cohesin at the borders of super-TADs.

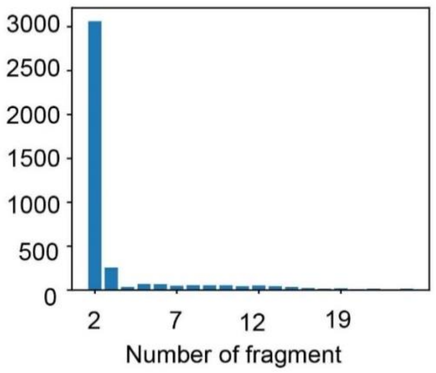

Supplementary Figure 3. The size distribution of the inter-TAD regions at fragment resolution. Most TADs are separated from each other by 2 restriction fragments (less than 400 bp). The sum of the fragments within the inter-TAD regions is 8.3% of the total number of fragments, reflecting a total length of the inter-TAD region of 7.1% of the non-repetitive genome.

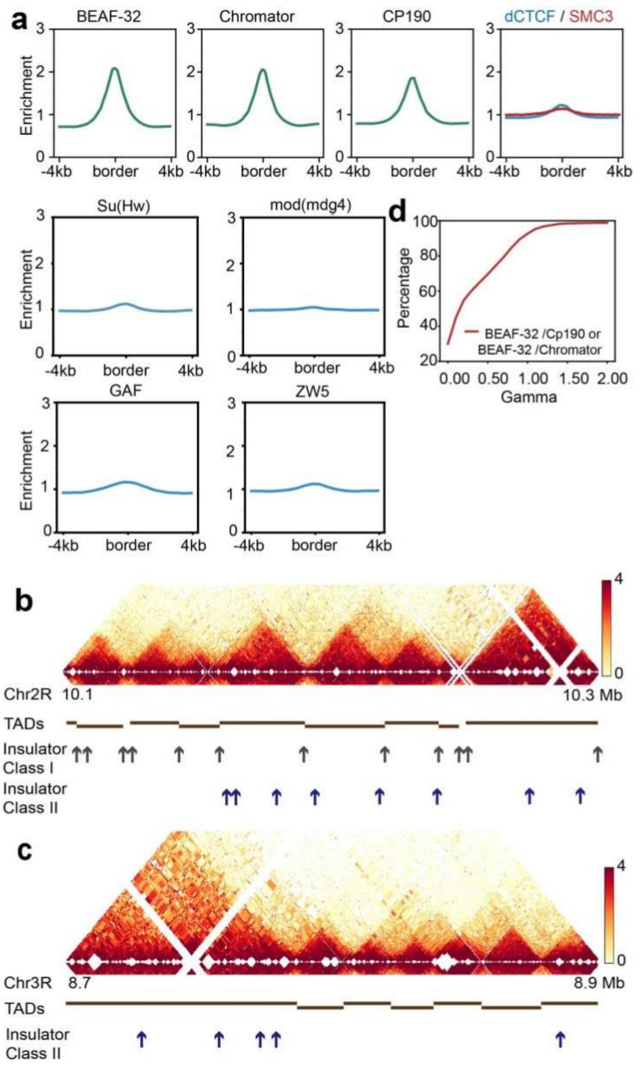

Supplementary Figure 4. TADs are demarcated by specific insulator proteins. (a) Extent of enrichment of insulator proteins studied in the modENCODE project at the TAD borders. (b,c) The spatial distribution of Class I (black arrows, as determined from Flybase) and Class II (blue arrows) with respect to the TAD borders. (d) The percentage of insulator pairs (BEAF-32/CP190 or BEAF-32/Chromator) that localize at TAD borders as a function of the gamma value used in the Armatus software to annotate the TADs, showing a strong enrichment over a wide range of gamma values.

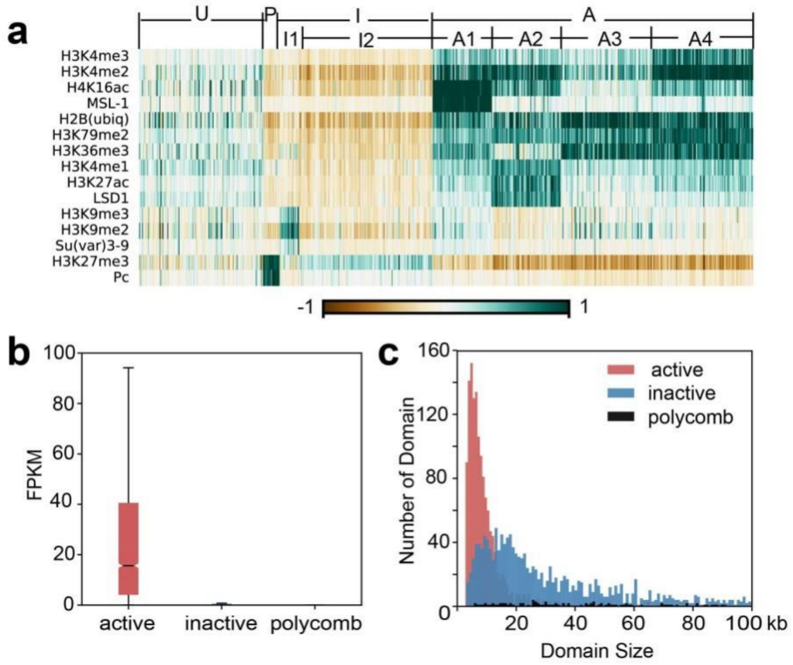

Supplementary Figure 5. Classification of the TADs according to the epigenetic modifications and non-histone proteins contained therein. (a) Classification of the TADs according to the enrichment of 15 histone modifications and non-histone chromosomal proteins within each TAD using k-means clustering. Eight different types of TADs, which could be further grouped into four major types, were identified. (b) The extent of transcription within the different types of TADs. (c) The size distribution of the different types of TADs.

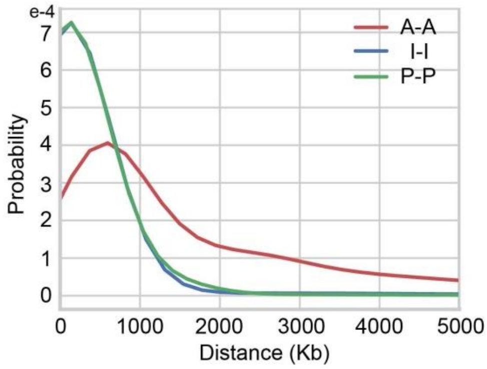

Supplementary Figure 6. The length distribution for regions exhibiting significant contact according to whether the loci are associated with active (A), inactive (I), or polycomb (P) chromatin.

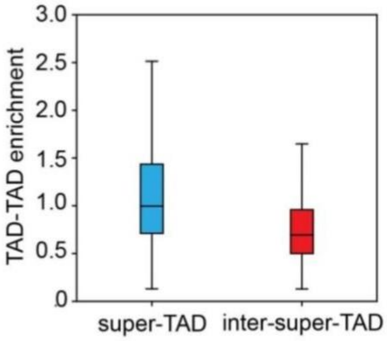

Supplementary Figure 7. Comparison of the frequency of TAD-TAD interactions within super-TAD and inter-super-TAD regions. The TADs within super-TADs contact each other more frequently than those within inter-super-TADs (Wilcoxon rank sum test p-value < 0.01).

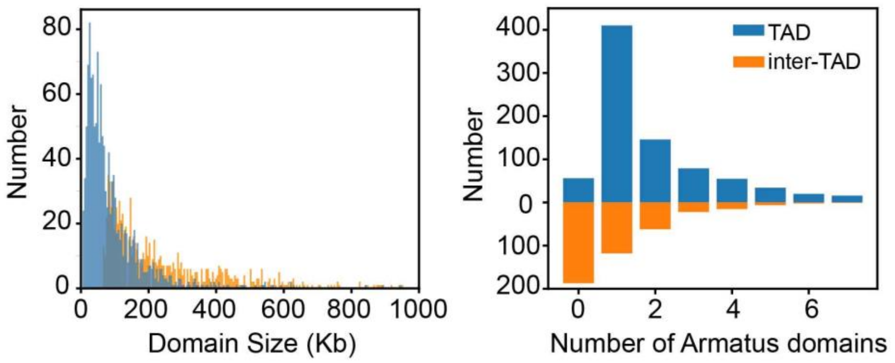

Supplementary Figure 8. Chromosome-wide identification of small TADs in human GM12878 cells using Armatus. The left panel shows the sizes of the domains identified previously[1] (orange) and those identified here using Armatus (blue) for chromosome 1. The right panel shows the number of the domains identified using Armatus within regions previously identified as TADs or inter-TADs[1].

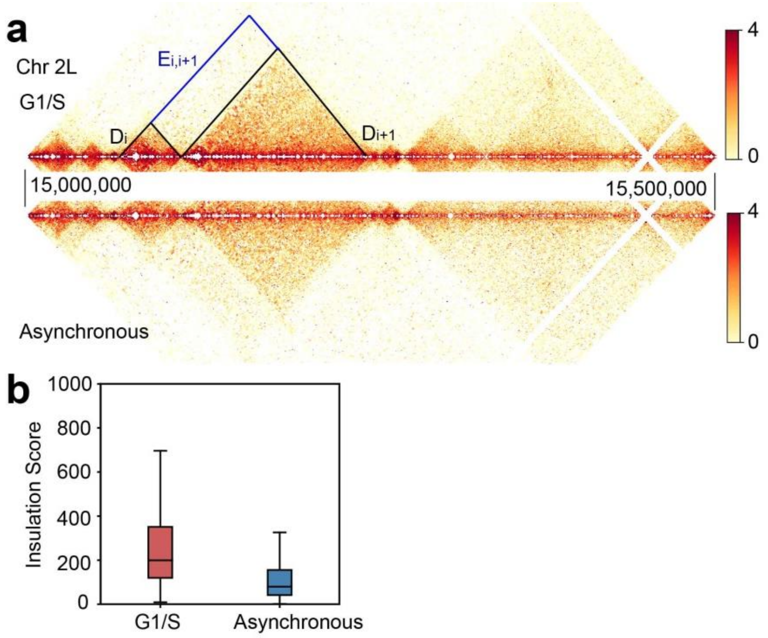

Supplementary Figure 9. The comparison between G1/S arrested and asynchronous cells. (a) A 500 kb region on chr2L showing the similarity of the TADs identified in the arrested or asynchronous cells. Also shown are the parameters used to calculate the insulation score: Di and Di+1 refer to the total interactions within the upstream and downstream TADs of a certain border, respectively, and Ei,i+1 refers to the interactions that include Di,i+1 and inter-TAD interactions between these two TADs. (b) Examination of the insulation score of the TAD borders shows that the TADs determined from the asynchronous cells are less well-defined than those of the G1/S synchronized cells.

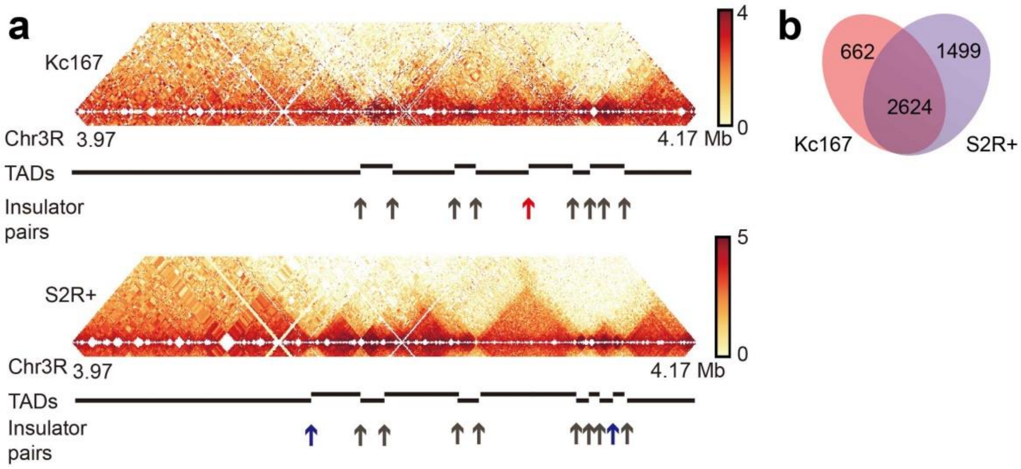

Supplementary Figure 10. The comparison of the insulator pair localizations and the TAD borders between in S2R+ and those in Kc167 cells. (a) The comparison of the locations of insulator pairs and corresponding TAD borders between those in S2R+ and in Kc167 cells illustrate that changes in the TAD border positions are associated with changes in the presence of the insulator proteins. The arrows show the positions of the insulator pair (BEAF-32/CP190 or BEAF-32/Chromator) with those colored black that are common between the cells, and those colored red (blue) that are specific to Kc167 (S2R+) cells. (b) 80% of the TADs found in Kc167 cells are conserved with those found in the S2R+ cells (see Materials and Methods). In addition, 68% of the pairs BEAF-32/CP190 or BEAF-32/Chromator localize to the TAD borders in Kc167 cells, and conversely, 70% of the borders localize to the binding sites of these pairs, showing a similar enrichment as found in S2R+ cells.

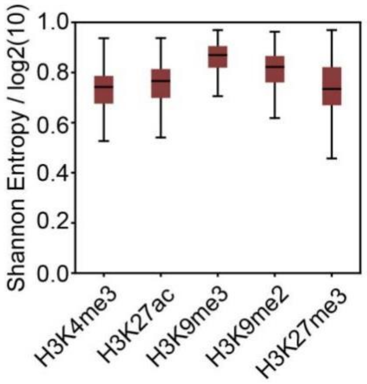

Supplementary Figure 11. The Shannon Entropy within each TAD. The calculation of the Shannon Entropy illustrates that there is an almost maximal degree of heterogeneity (a value of 1) of the epigenetic markers within the TADs.

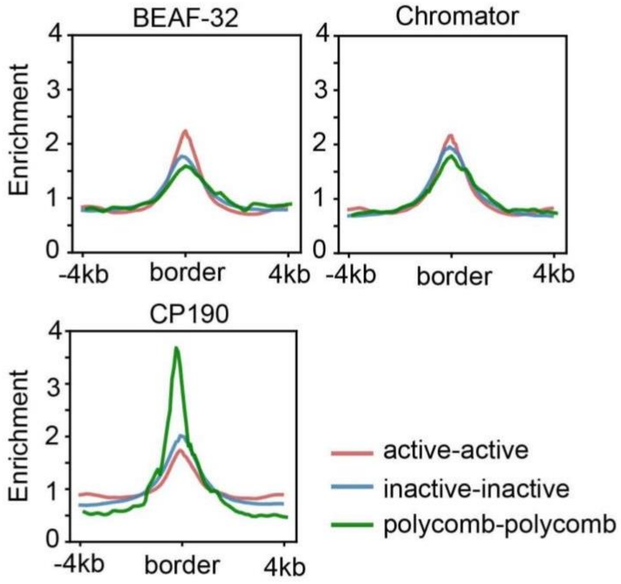

Supplementary Figure 12. The distribution of the insulator proteins near borders of TADs as a function of the chromatin type within the adjacent TADs. Each of the insulator proteins, BEAF-32, Chromator, and CP190 is enriched at all borders, regardless of the chromatin state within the TAD. The significance of the greater enrichment of CP190 at the Polycomb-Polycomb borders, which accounts for only 19 out of 3866 (0.5%) of all shared borders, requires more detailed examination.

Supplementary References

1. Rao, S.S. et al. A 3D map of the human genome at kilobase resolution reveals principles of chromatin looping. *Cell* **159**, 1665-80 (2014).
2. Ulianov, S.V. et al. Active chromatin and transcription play a key role in chromosome partitioning into topologically associating domains. *Genome Res* **26**, 70-84 (2016).
3. Imakaev, M. et al. Iterative correction of Hi-C data reveals hallmarks of chromosome organization. *Nat Methods* **9**, 999-1003 (2012).
4. Filippova, D., Patro, R., Duggal, G. & Kingsford, C. Identification of alternative topological domains in chromatin. *Algorithms Mol Biol* **9**, 14 (2014).
5. Dixon, J.R. et al. Topological domains in mammalian genomes identified by analysis of chromatin interactions. *Nature* **485**, 376-80 (2012).
6. Cubenas-Potts, C. et al. Different enhancer classes in Drosophila bind distinct architectural proteins and mediate unique chromatin interactions and 3D architecture. *Nucleic Acids Res* **45**, 1714-1730 (2017).
