# Supplementary material for: Sub-kb Hi-C in D. melanogaster reveals conserved characteristics of TADs between insect and mammalian cells
Source: Nat Commun. 2018 Jan 15;9:188. doi: 10.1038/s41467-017-02526-9 (PMC5768742; doi:10.1038/s41467-017-02526-9)
Supplement: Supplementary file 2 — Description of Additional Supplementary Files [file 41467_2017_2526_MOESM2_ESM.pdf]

Description of Additional Supplementary Files

File Name: Supplementary Data 1
Description: Super-TADs in G1/S-arrested S2R+ cells.

File Name: Supplementary Data 2
Description: TADs in G1/S-arrested S2R+ cells.
